# The HBx Oncoprotein of Hepatitis B Virus Deregulates the Cell Cycle by Promoting the Intracellular Accumulation and Re-Compartmentalization of the Cellular Deubiquitinase USP37

**DOI:** 10.1371/journal.pone.0111256

**Published:** 2014-10-27

**Authors:** Nehul Saxena, Vijay Kumar

**Affiliations:** Virology Group, International Center for Genetic Engineering and Biotechnology, Aruna Asaf Ali Marg, New Delhi, India; Drexel University College of Medicine, United States of America

## Abstract

The HBx oncoprotein of hepatitis B Virus has been accredited as one of the protagonists in driving hepatocarcinogenesis. HBx exerts its influence over the cell cycle progression by potentiating the activity of cyclin A/E-CDK2 complex, the Cyclin A partner of which is a well-known target of cellular deubiquitinase USP37. In the present study, we observed the intracellular accumulation of cyclin A and USP37 proteins under the HBx microenvironment. Flow cytometry analysis of the HBx-expressing cells showed deregulation of cell cycle apparently due to the enhanced gene expression and stabilization of USP37 protein and deubiquitination of Cyclin A by USP37. Our co-immunoprecipitation and confocal microscopic studies suggested a direct interaction between USP37 and HBx. This interaction promoted the translocation of USP37 outside the nucleus and prevented its association and ubiquitination by E3 ubiquitin ligases - APC/CDH1 and SCF/β-TrCP. Thus, HBx seems to control the cell cycle progression via the cyclin A-CDK2 complex by regulating the intracellular distribution and stability of deubiquitinase USP37.

## Introduction

The momentum of cell cycle is governed by the temporal synthesis, maintenance and degradation of cell cycle regulators. A plethora of E3 ubiquitin ligases and deubiquitinases (DUBs) capable of reversing ubiquitination, are now considered integral to the regulation of cell cycle [1]–[4]. So far fifteen different DUBs including USP2, USP3, USP7, USP13, USP17L2, USP19, USP28, USP37, USP39, USP44, USP50, COP9 sinnalosome subunit 5 (CSN5), BRCA1 associated protein-1 (BAP1), Cylindromatosis protein (CYLD) and Ovarian tumor domain containing subunit 6B (OTUD-6B) have been implicated in cell cycle regulation [5]. Particularly, USP37 which belongs to the ubiquitin-specific protease family of DUBs, regulates cell cycle by antagonizing the activity of APC/CDH1 complex during the G1/S boundary, S and G2 phases to stabilize its substrate Cyclin A [6]. The USP37 gene is transcriptionally activated by transcription factor E2F followed by its translation during the G1/S boundary of cell cycle. The USP37 protein becomes fully functional upon its Cyclin A/CDK2-mediated phosphorylation at Ser-628 residue [6] and remains active throughout the S phase upto G2/M boundary. Apparently, the degradation of USP37 occurs in a bi-phasic manner. At the G2/M boundary, polo like kinase 1 (Plk1)-dependent phosphorylation of serine residues in *DSGXXS* consensus motif makes USP37 vulnerable to Skp1-Cullin1-F-box ubiquitin ligase/beta-transducin repeat containing protein complex (SCF/β-TRCP)-mediated ubiquitination and proteasomal degradation [7]. Also, during the M phase, upon depletion of Cyclin A and subsequent disappearance of CDK2 activity, the residual un-phosphorylated USP37 undergoes proteasomal degradation following its APC/CDH1-mediated KEN-box dependent ubiquitination [6]. Apart from its physiological relevance, USP37 is also reported to play an important role in cancer. For instance, increased USP37 expression is correlated with poor prognosis in non-small cell lung cancer [8]. It also confers resistance to Acute promyelocytic leukemia cells against arsenic trioxide and all-trans retinoic acid treatment by preserving the PLZF-RARA (promyelocytic leukemia zinc finger and retinoic acid receptor alpha) fusion protein [9]. Ambiguously, the transcription of USP37 is suppressed in medulloblastoma cells through the activity of RE1 silencing transcription factor to prevent the USP37-mediated stabilization of the cyclin-dependent kinase inhibitor p27, which is known to act as a negative regulator of cell cycle [10].

The HBx oncoprotein of hepatitis B virus (HBV) is a multifaceted transactivator protein that can induce growth promoting signaling pathways, inhibit DNA damage response, stabilize cell cycle regulators and destabilize inhibitors of cell cycle to favor unchecked cellular proliferation and create an ambience conducive for the development of hepatocellular carcinoma (HCC) in the host [11]. Under the HBx microenvironment, the Cyclin E/A-CDK2 complex is constitutively activated to hyperphosphorylate and inactivate pRb to accelerate the G1/S phase transition by activating E2F transcription factor [12]. Deviating from normalcy, HBx also stabilizes and maintains Cyclin A protein levels throughout the cell cycle [13] in contrast to its usual degradation during mitosis by anaphase promoting complex and its adaptor CDC20 homologue 1 (APC/CDH1) [14]. Thus, a premature surge in Cyclin A/CDK2 activity [13] and downregulation of CDH1 protein levels [15] under the HBx microenvironment, may create an ambience conducive for enhanced USP37 activity. Akin to this, earlier studies illustrating the close association of USP37 with cell cycle regulation [6], [10] and tumorigenesis [8]–[10] makes USP37 a likely target that could be manoeuvred by HBx to orchestrate HCC development.

The present study revealed the intracellular accumulation of USP37 under the HBx microenvironment resulting in the stabilization of its target and key cell cycle regulator cyclin A. The stabilization of USP37 and Cyclin A and consequent increase in cyclin-CDK2 activity apparently led to deregulation of the cell cycle. Further, we observed that HBx interacted with USP37 and chaperoned it out of nucleus to prevent its ubiquitination and degradation by E3 ubiquitin ligases.

## Materials and Methods

### DNA recombinants

The HA-tagged HBx expression construct was developed by cloning HBx gene in pSG5 vector (Stratagene) [16]; X0-MBP was obtained by cloning HBx gene into a modified pMal-Xa vector (NEB) [17] and the shRNA against HBx (X-E) was obtained by cloning 5′-phosphorylated and annealed oligonucleotides corresponding the siRNA sequence targeting transactivation domain of HBx into pSilencer 1.0-U6 (Ambion, USA) [18]. X-E shRNA construct was validated by monitoring the expression of X0-GFP construct upon its co-transfection with Scrambled (Sc) shRNA or X-E shRNA construct, using bright field and fluorescent microscopy (**Figure S2D in file S1**). X0-GFP recombinant construct was received from Addgene. The recombinants HA-CDH1, Flag-USP37, and Flag-USP37-DUB-Dead were kindly provided by Dr. Vishwa Mohan Dixit (Genentech) [6]; X0-NESM-GFP construct was a kind gift from Dr. Xin Wei Wang (National Institutes of Health, Bethesda, Maryland, US) [19]; FLAG-Emi1 construct was kindly provided by Dr. Anindya Dutta (University of Virginia, Charlottesville, VA, US) [20]; pCDNA3-β-TRCP and pCDNA3-ΔF-β-TRCP were kindly provided by Dr. Kei-ichi Nakayama (Department of Molecular and cellular biology, Kyushu university, Japan) [21]; wild-type E2F1 (pCMV-E2F1) and its transactivation defective mutant pCMV-E2F1-1-374 (ΔC) from obtained as kind gifts from Dr Xin Lu (Ludwig Institute for Cancer Research, Cambridge, UK) [22] and Myc-ubiquitin construct was kindly provided by Dr. Michael MC Lai (Institute of Molecular Biology, Academia Sinica, Taipei, Taiwan) [23].

### Antibodies

USP37 antibody was acquired from Proteintech; Flag (1∶4000) antibody was acquired from Sigma; β-TRCP antibody was acquired from Cell Signaling; Emi1 (1∶500), GAPDH, CDC6, phospho-CDC6, Geminin, β-catenin, Histone H1, Myc-tag, β-TRCP/HOS, HBx, Myc, Ubiquitin, HA, Anti-rabbit and Anti-mouse–HRP conjugates were acquired from Santa Cruz Biotechnology; CDH1 from Abcam and phospho-Serine (4A4) antibody was acquired from Millipore. Anti-mouse-Alexa-Fluor-488 and Anti-rabbit Alex-Fluor-594 were obtained from Life technologies. All the primary antibodies were used at a dilution of 1∶1000 for western blotting and 1∶250 dilution for confocal microscopy unless mentioned otherwise. The secondary antibodies were used at dilution of 1∶5000 for western blotting and 1∶1500 for confocal microscopy.

### Cell Culture and transfection

Human hepatoma Huh7 cells and Immortalized human hepatocytes IHH cells were received as kind gifts from Dr. Aleem Siddiqui (University of Colorado, Denver) [16] and Dr. F. Danniel (Institut National de la Santé et de la Recherche Médicale Unite 481, Universite Paris 7, Paris, France) [17], respectively. Human embryonic kidney Cells (HEK293T) and Human bone osteosarcoma epithelial (U2OS) cells were obtained from ATCC. All the cell lines were maintained in DMEM supplemented with 10% FBS at 37°C in 5% CO_2_. Transfection was carried out in culture dish with indicated plasmids by Lipofectamine (Invitrogen) according to the manufacturer’s instructions. In general, a total of 1 ug DNA was transfected per well of 12 well dish; 2 ug DNA was transfected in 60 mm dish and 5 ug DNA was transfected in 100 mm dish, unless mentioned otherwise. During co-transfection equal ratio of each plasmid DNA was added to maintain this stoichiometry. shRNA (Scrambled or X-E) and HBx constructs (HA-HBx or HBx-GFP) were transfected at a ratio of 2∶1. IHH cells were synchronized by serum starvation for 72 h followed by release in 10% DMEM for designated time. Where indicated cells were treated with 20 µM MG132 for 6 h (Calbiochem); 300 µg Cycloheximide for indicated time intervals (Amresco); 100 nM Leptomycin B (Sigma) for 4 h; 10 µM CDK2 inhibitor II compound 3 (Calbiochem) for 6 h, 100 µM PLK1 inhibitor SBE 13 hydrochloride (Sigma-Aldrich) for 8 h and 0.1% (v/v) Methyl methane sulphonate (97% w/v) for 30 min.

### Brd-U incorporation assay

5-Bromo-2′-deoxy-uridine detection and labeling kit I (Roche) was used to perform Brd-U incorporation assay as per the manufacturer’s protocol for cells grown on coverslips.

### Co-immunoprecipitation assay and Western Blotting

Co-imunoprecipitation was performed using Thermo-Fischer Pierce Co-immunoprecipitation Kit as per manufacturer’ protocol. The beads were boiled in 2x lysis buffer to release the immuno-complex. The samples were resolved on SDS-PAGE gel were transferred onto the nitro-cellulose membrane (MDI). The blots were blocked with 5% blocking at 37°C for 1 h followed by incubation with primary antibody overnight at 4°C. Subsequently, the blots were washed thrice for 5 minutes each with 1x PBST (Phosphate-buffered saline with 1% Triton-100) and were then incubated with secondary antibody for 2 h at 37°C. The blots were then washed thrice for 5 min each with 1x PBST. The blots were developed on X-ray films (Amersham or Kodak) after incubation with ECL reagent (Immunocruz, Santa Cruz biotechnology).

### Cytoplasmic-Nuclear fractionation

Huh7 cells transfected with desired constructs (5 ug DNA of Vector, HBx-GFP and HBx-NESM-GFP, respectively in 100 mm dishes or 3 ug vector with 2 ug Scrambled shRNA, 1 ug HBx with 2 ug Scrambled shRNA equalized with 2 ug of vector or 1 ug HBx with 2 ug X-E shRNA equalized with 2 ug of vector, respectively in 100 mm dishes) were harvested 48 hours post-transfection. Cells were incubated in buffer A [10 mM Hepes (pH-7.9), 10 mM KCl, 0.1 mM EGTA, 0.1 mM EDTA, 1 mM DTT, 1 mM PMSF and 1x PIC) for 15 min at 4°C. 10% NP40 was added to cells suspended in Buffer A and vortexed vigorously for 15 sec. The nuclear (pellet) and cytoplasmic fractions (supernatant) by centrifuging at 13,000 r.p.m. at 4°C for 30 seconds. Nuclear fraction was resuspended in buffer B [20 mM Hepes (pH, 7.9), 1 mM EDTA, 1 mM EGTA, 1 mM DTT, 400 mM NaCl, 20% Glycerol (V/V), 1 mM PMSF and 1x PIC] and incubated at 4°C for 45 minutes on a Nutator. After centrifugation at 5000 r.p.m. for 5 min at 4°C, the nuclear fraction (supernatant) was collected in separate tube. Protein was quantified by using Bradford’s Dye (BioRad), electrophoretically separated on SDS-PAGE gel and western blotted with desired antibodies.

### Flow Cytometry

IHH cells 24 h post-transfection were starvedfor 72 h and then werestimulated with serum for the indicated timeperiods. After washing with PBS, cells were fixed at 4°C with 70% ethanol, washed again with PBS and incubated in PBS containing 100 µg/ml RNaseA at 37°C for 30 min. After staining with propidium iodide overnight at 4°C the DNA content of cells was analyzed with a FACS Calibur Flow Cytometer (BD Biosciences) using the Cell Quest software. The cell synchronization was ascertained by monitoring the Cyclin E, Cyclin A and p27 levels in cell population harvested at various time points post-serum stimulation in parallel with FACS analysis (**Figure S1A in file S1**).

### Immunoflorescence microscopy

Cells grown on cover slips were fixed with 2% paraformaldehyde for 20 min, permeabilized with 0.4% Triton X-100 for 20 min and then blocked with PBS containing 0.5% bovine serum antigen for 1 h at room temperature. Immunostaining was performed with appropriate primary antibody followed by incubation with corresponding fluorescent-labelled secondary antibody. Nuclei were stained with DAPI and mounted using prolong-Antifade (Invitrogen). Photomicrographs were captured at 60x magnification in Nikon A1R confocal microscope. Images were processed and co-localization co-efficient were determined using NIS Elements AR 3.0 software (Nikon).

### *In-vitro* ubiquitination assay

Cells transfected with regulatory plasmid or control plasmid; bait DNA and Myc-Ubiquitin construct (A total of 6 ug DNA was transfected in a 100 mm dish) were treated with 20 µM MG132 6 h before harvesting and lysed in 2x cell lysis buffer. After incubation with desired antibody, The samples immunoprecipitated with the indicated antibody using the Thermo-Fischer Pierce Co-immunoprecipitation Kit as per manufacturer’ protocol, electrophoretically separated on SDS-PAGE gel, transferred onto the nitro-cellulose membrane (MDI) and immunoblotted with anti-ubiquitin antibody.

### MTT Assay

Huh7 cells overexpressing indicated plasmids or given indicated treatments were incubated with 200 ul of MTT solution (1 mg/ml 3-(4,5-dimethylthiazol-2-yl)-2,5-diphenyltetrazolium bromide (MTT) in 1 ml DMEM without Phenol Red) at 37°C for 30 min in dark. The media was discarded and cells were extracted in 1 ml DMSO and the absorbance was measured at 560 nm.

### Quantitative real-time RT-PCR

Total RNA was isolated from cells using TRIzol reagent as per manufacturer’s instructions (Invitrogen). Reverse transcriptase–PCR (RTPCR) was performed with M-MuLV reverse transcriptase (Fermentas) according to the manufacturer’s guidelines. Prepared cDNA samples were amplified using specific primers (Table S1 in File S1) and analyzed by quantitativereal-time PCR with 2x Brilliant III SYBR Green qPCR Master Mix (BioRad) using Step One plus Real Time PCR System Thermal cycling Block (Applied Biosystems). Each sample was assessedin sets of triplicate. Relative mRNA levels were normalized to GAPDH mRNA and calculated using thecomparative threshold cycle method (2^−ΔΔCt^) [26].

### Statistical analysis

Data are expressed as mean ± S.E. Statistical significance was calculated using Student’s t test. P values<0.05 were considered significant.

## Results

### Intracellular accumulation of Cyclin A in the presence of HBx is dependent on USP37

**Figure 1:**
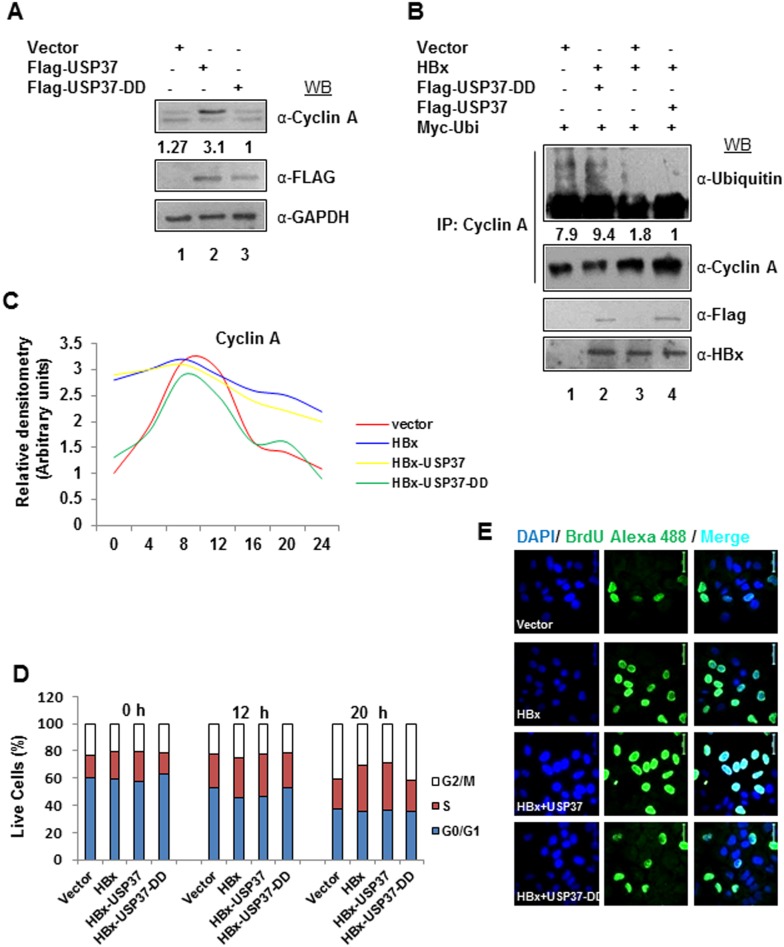
USP37 and HBx act in synergy to deregulate cell Cycle. (A) Huh7 cells were transfected with Vector, Flag-USP37 and Flag-USP37-DD construct and the levels of USP37 protein were measured by western blotting (WB). (B) Ubiquitination assay was performed with lysates from cells transiently expressing Vector, HA-HBx, Flag-USP37, Flag-USP37-DD and Myc-Ubi as indicated (Cells in 100 mm dish were transfected with 4 µg Vector and 2 µg Myc-ubiquitin; 2 µg Vector, 2 µg HA-HBx and 2 µg Myc-ubiquitin; 2 µg Flag-USP37, 2 µg HA-HBx and 2 µg Myc-ubiquitin or 2 µg Flag-USP37-DD, 2 µg HA-HBx and 2 µg Myc-ubiquitin) and treated with 20 µM MG132 for 6 h, by immunoprecipitating Cyclin A. Immuno-complexes were eluted and western blotted with α-Ubiquitin antibody. (C) Cyclin A expression was chased in IHH cells transiently transfected with Vector, HA-HBx, Flag-USP37 and Flag-USP37-DD as indicated and harvested at indicated time intervals post 72 hrs serum starvation. (D) IHH cells transfected with Vector control, HA-HBx, Flag-USP37 and Flag-USP37-DD as indicated, were synchronized in G0/G1 phase by Serum starvation followed by harvesting at indicated time points. Cells in different phases of cell cycle were analyzed by flow cytometry. Values are represented as bar diagrams (E) Brd-U incorporation assay was carried out in Huh7 cells transfected with Vector control, HA-HBx, Flag-USP37 and Flag-USP37-DD as indicated, by incorporating BrdU followed by staining with antibody against BrdU and counterstaining with DAPI to observe actively replicating cells as seen in the representative confocal images. Scale bar represents 50 µm.

HBx has been attributed to deregulate cell cycle by multiple mechanisms. Among these, HBx helps maintaining the Cyclin A levels and potentiates cyclin A-CDK2 complex activity to accelerate S phase entry without cellular catastrophy [12], [13]. As USP37 is well known to deubiquitinate and stabilize cyclin A, we wondered if the sustained levels of Cyclin A and consequent increase in the CDK2 activity under the HBx micro-environment was mediated by USP37. We used two expression constructs of USP37-full length (USP37) and DUB dead mutant (USP37-DD) to evaluate the stability of Cyclin A in Huh7 cells. As reported earlier [6], we observed a decline in the levels of Cyclin A in the presence of USP37-DD but not USP37 (**Figure 1A**). Next, we examined the ubiquitination status of Cyclin A in the presence of HBx and both the USP37 recombinants. As shown in **Figure 1B**, Cyclin A ubiquitination was relatively lower in the presence of HBx alone or HBx along with USP37 (lanes 3,4). However, USP37-DD relieved the restraint on Cyclin A ubiquitination (**Figure 1B** **, lane 2**). Analysis of Cyclin A expression in the synchronized population of IHH cells revealed that while its levels were maintained in HBx transfected cells upto 24 h post serum stimulation, the Cyclin A levels started declining 16 h post serum stimulation in control cells (**Figure 1C**). We then monitored the levels of Cyclin A in IHH cells co-transfected with HBx-USP37 and HBx-USP37-DD. We observed that while HBx and USP37 co-transfection helped in maintaining the levels of Cyclin A, HBx and USP37-DD co-transfection caused a decline in Cyclin A levels at 16 h post serum stimulation (**Figure 1C**). As expected, transfection with USP37 alone conferred stability to CyclinA when compared to USP37-DD mutant (**Figure S1B in file S1**). These results indicated that USP37 plays a niche role in HBx-mediated CyclinA homeostasis. Further, FACS analysis of these cells revealed that majority of cells transfected with HBx alone or HBx and USP37 circulated in S phase irrespective of time of serum stimulation (0–20 h) while the vector and HBx-USP37-DD transfected cells showed some variation in the distribution of cells (**Figure 1D**). This result indicated that USP37 along with HBx hastened the S phase entry of cells. Further, MTT assay of these cells ruled out the possibility of cell cycle arrest as the HBx-USP37 co-transfected cells showed higher viability just as c-Myc transfected cells compared to HBx-USP37-DD mutant co-transfected, MMS treated cells or control cells (**Figure S1C in file S1**). As Cyclin A expression is critical for DNA synthesis and S phase progression, we reasoned that cells with stable Cyclin A expression might show better 5-bromo-2′-deoxy-uridine (Brd-U) incorporation. Not surprisingly, the number of Brd-U positive cells was higher (at least ∼2 fold) in HBx alone and HBx-USP37 co-transfected cells compared to those transfected with HBx-USP37-DD construct or control cells (**Figure 1E**). Interestingly, the USP37 over-expressing cells also exhibited higher Brd-U incorporation as compared to the cells over-expressing USP37-DD construct (**Figure S1D in file S1**). These observations implicated USP37 as a crucial player in the HBx-mediated deregulation of cell cycle.

### HBx augments the expression of USP37

**Figure 2:**
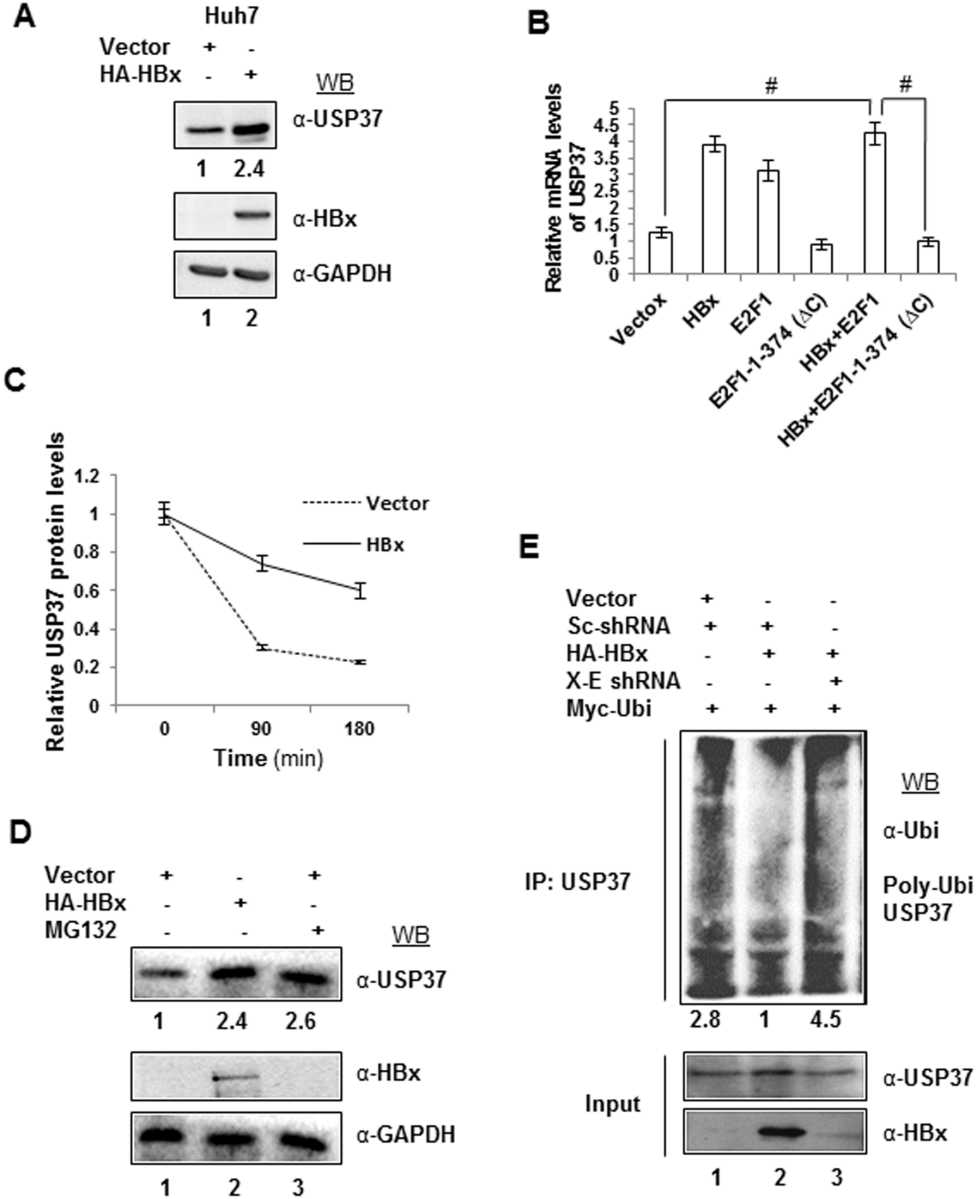
HBx upregulates USP37 mRNA and protein levels. (A) Cell lysates from Huh7 cells transfected with Vector control or HA-X0 construct were run on SDS-PAGE gel and were blotted for USP37 and normalized against GAPDH (B) Total RNA was isolated and USP37 mRNA levels were measured by RT-qPCR using specific primers (Table S1 in File S1) in Huh7 cells overexpressing Vector, HA-HBx, E2F1 and E2F1-1-374-ΔC as indicated. Data (bar diagrams) are shown as mean ± SD of three independent observations # represents statistically significant difference of p<0.001. (C)Stability of USP37 protein was monitored in Huh7 cells transfected with vector or HA-HBx then treated with 20 µg/ml cycloheximide for the indicated durations. Change in Endogenous USP37 protein levels were detected by western blotting using antiUSP37 antibody as indicated in line graph. GAPDH was used as a control. Data (line graph) are shown as mean ± SD of three independent observations. (D) Cell extracts from Huh7 cells transfected with Vector or HA-X0 as indicated, were treated with 20 µM of MG132 for 6 hrs and western blotted for USP37 protein and normalized with GAPDH. (E) Ubiquitination assay was performed by immunoprecipitating USP37 from cell lysates from Huh7 cells transiently transfected with Vector, HA-HBx, X-E and Myc-Ubiquitin as indicated (1 ug vector, 2 ug scrambled shRNA and 2 ug Myc-ubiquitin; 1 ug HA-HBx, 2 ug scrambled shRNA and 2 ug Myc-ubiquitin or 1 ug HA-HBx, 2 ug X-E shRNA and 2 ug Myc-ubiquitin were transfected in 100 mm dishes) and treated with MG132, as mentioned above and western blotting the immino-complexes with anti-Ubiquitin Anitibody.

Since USP37 appeared to be a mediator of HBx activity, we next investigated the influence of HBx on USP37. We found that ectopic expression of HBx resulted in an increase in the levels of USP37 protein both in Huh7 and IHH cells (**Figures 2A and S** **2A**). The HBx-dependent up-regulation of USP37 could be seen even in non-hepatic cells such as HEK293T and U2OS (**Figure S2B** and **S2C in file S1**). As USP37 is already known to be transcribed in an E2F1-dependent fashion [6] and E2F1 is transcriptionally up-regulated by HBx [27], we next analyzed the expression of USP37 mRNA in the presence of HBx. As shown in **Figure 2B** **,** there was a marked increase in USP37 transcription (p<0.001) in the presence of HBx or E2F1 alone or HBx along with E2F1. Further, the HBx-dependent expression of USP37 mRNA was inhibited in the presence of a transactivation domain mutant of E2F1-1-374 (ΔC) [24] (**Figure 2B**) thereby, reflecting on the dependence of HBx on E2F1 in trans-activating the USP37 gene.

The rise in protein expression can often be correlated to an upsurge in transcript levels as previously observed in case of the replication licensing factor CDC6 [27]. Surprisingly, a minimal or no change was observed in the USP37 protein levels upon transfection of cells with either E2F1 alone or E2F1-1-374 (ΔC) along with HBx. Note that CDC6 protein level that remained static in HBx or HBx and E2F1 co-expressing cells but was down-regulated after co-transfecting HBx and E2F1-1-374 (ΔC) (**Figure S2E in file S1**). These observations thereby indicated the role of other mechanisms in USP37 protein up-regulation. As the observed increase in USP37 levels in the presence of HBx could be due to enhanced protein stability, we measured the half-life of USP37 by blocking the *de-novo* protein synthesis with cycloheximide. As shown in **Figure 2C**, there was a marked improvement in USP37 stability under these conditions. Since USP37 is degraded by the proteasomal machinery after ubiquitination by SCF/β-TrCP and APC/CDH1 complex, we wondered if USP37 could escape proteasomal degradation machinery in the presence of HBx. The levels of USP37 protein were monitored after treating the cells with proteasomal inhibitor MG132. Not surprisingly, the MG132-treated cells showed a marked increase in USP37 levels equivalent to HBx transfected cells, when compared to untreated cells (**Figure 2D**). Thus, HBx seemed to facilitate the accumulation of USP37 by preventing its proteasomal degradation. In support, ubiquitination assay confirmed that HBx interfered with USP37 ubiquitination and the effect could be reversed by using sh-RNA against HBx (**Figure 2E**).

### HBx differentially regulates E3 ubiquitin ligases to stabilize USP37

**Figure 3:**
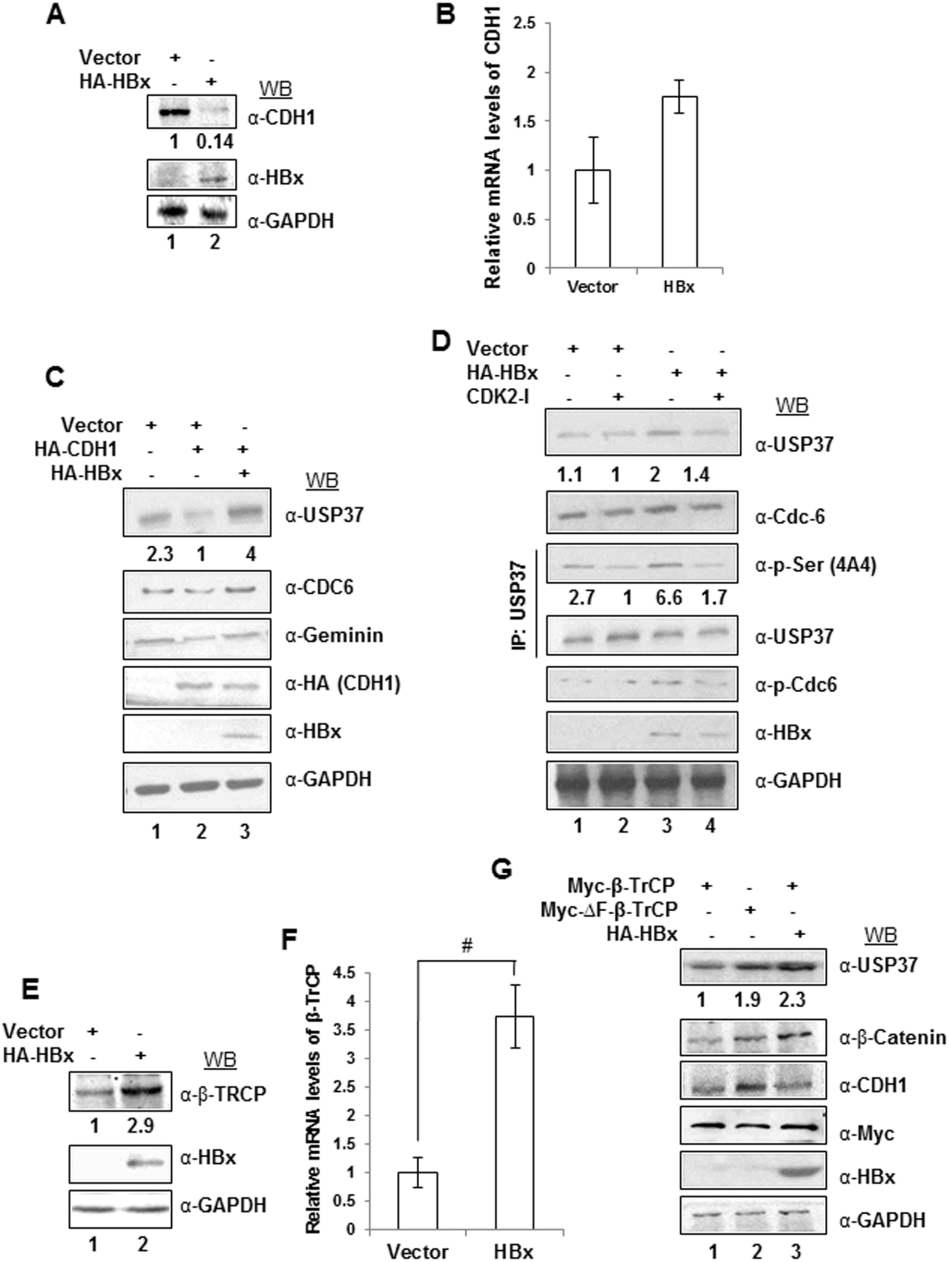
HBx differentially regulate CDH1 and β-TrCP to circumvent USP37 downregulation. (A) Cell extracts of Huh7 cells ectopically expressing Vector or HA-HBx were western blotted with anti-CDH1 antibody and normalized with GAPDH. (B) Relative m-RNA levels of CDH1 in Vector or HA-HBx transfected cells, were measured by performing qRT-PCR with primers mentioned in Table S1 in File S1. GAPDH was used as control. Data (bar diagrams) are shown as mean ± SD of three independent observations. (C) Cell lysates from cells expressing vector alone; co-expressing vector and HA-CDH1 and HA-CDH1 and HA-X0 were western blotted for USP37, CDC6, Geminin and GAPDH with indicated antibodies. (D) Untreated and CDK2 Inhibitor II compound 3 (10 µM for 6 h) treated Vector or HBx transfected cell extract were western blotted for USP37, CDC6 and GAPDH. Immunoprecipitation assay was performed using USP37 antibody with cell lysates from untreated or CDK2 inhibitor II compound 3 (10 µM for 6 h) treated Vector or HBx transfected cells. The immune-complexes were blotted with phospho-serine and USP37 antibody. Total cell lysates were also western blotted with phospho-CDC6 (Ser-54) and GAPDH antibody. (E) Cell lysates from cells expressing Vector or HA-HBx were western blotted with anti-β-TrCP antibody and normalized with GAPDH. (F) Relative m-RNA levels of β-TrCP in Vector or HA-HBx transfected Huh7 cells, were measured by performing qRT-PCR with primers mentioned in Table S1 in File S1. GAPDH was used as control. Data (bar diagrams) are shown as mean ± SD of three independent observations # represents statistically significant difference of p<0.001. (G) Myc-β-TrCP, Myc-ΔF-β-TrCP and HA-HBx transfected huh7 cells (as indicated) were lysed and lysates was separated on SDS-PAGE gel western blotted for USP37, β-catenin and CDH1. GAPDH was used as a loading control.

USP37 is degraded in a biphasic fashion by two well established E3 ubiquitin ligase complex -SCF/β-TrCP during the G2/M phase and APC/CDH1 from mitosis to early G1 phase. Having established the role of HBx in USP37 gene expression and protein stability, we next monitored the regulation of CDH1 and β-TrCP in HBx microenvironment. Since, HBx is known to interfere with the ubiquitination of CDC6 by negatively regulating protein levels of CDH1 [27], we wondered if HBx similarly conferred protection to USP37 from CDH1-mediated degradation. Not surprisingly, CDH1 levels were down-regulated in the presence of HBx (**Figure 3A**). Incidentally, HBx had only a marginal effect on the basal CDH1 transcripts (**Figure S3A in file S1**). Further, we found that ectopic expression of CDH1 led to decreased levels of USP37 protein (**Figure 3B**). Conversely, HBx rescued USP37 from CDH1-mediated down-regulation similar to CDC6 but not the other CDH1 substrate, Geminin [28] (**Figure 3C**). Consistently, the HBx-mediated protection of USP37 from CDH1 was attenuated by RNA interference against HBx (**Figure S3B in file S1**).

Early mitotic inhibitor 1 (Emi1) has been reported to act as psuedosubstrate of CDH1 to provide stability to CDH1 substrate by interfering with CDH1-substrate association [29]. Addressing the possibility of Emi1 subjugating the activity of CDH1, USP37 protein levels were monitored after co-expressing Emi1 and HBx. Ironically, no change in the USP37 protein levels was observed either by Emi1 alone or along with HBx (**Figure S3C in file S1**).

As HBx is known to potentiate Cyclin-associated CDK2 activity which endows stability to CDC6 by protecting it against APC/CDH1 catalyzed ubiquitination [27], we next investigated the role of CDK2-mediated phosphorylation in the stabilization and accumulation of USP37 protein in the presence of HBx. We observed that just as CDC6, the levels of USP37 protein and phosphorylated USP37 decreased in the presence of CDK2 inhibitor (**Figure 3D**). Interestingly, the USP37 protein and phosphorylated USP37 protein levels remained static in the vector transfected cells irrespective of CDK2 inhibitor treatment thereby highlighting the significance of CDK2 mediated phosphorylation in HBx-mediated accumulation of USP37(**Figure 3D**). Thus, the down-regulation of CDH1 and CDK2-dependent phosphorylation seem responsible for conferring intracellular stability to USP37 in HBx microenvironment.

We next sought to understand the involvement of other E3 ubiquitin ligase, β-TrCPin the regulation of USP37 levels by HBx. We found that, in stark contrast to CDH1, β-TrCP was upregulated in the presence of HBx, both at the protein and transcript levels (**Figure 3E and 3F**). As reported earlier [7], β-TrCP overexpression led to the downregulation of USP37 protein levels in the cell (**Figure S3D in file S1**). While the ectopic expression of ΔF-box deletion mutant of β-TrCP [23] could rescue the levels of its substrates like USP37, β-catenin [23] and Iκβα [30], HBx over-expression also conferred stability to β-catenin similar to USP37 (**Figure 3E**). Further, RNA interference against HBx counteracted its ability to vanquish β-TrCP catalyzed down-regulation of USP37 (**Figure S3E in file S1**).

Plk1-mediated phosphorylation of USP37 plays an important role in its recognition by SCF-β-TrCP [7]. Besides, several line of evidence also indicate that PLK1 can be activated in the presence of HBx [31]. Hence, we next investigated whether USP37 was insulated from PLK1 activity in the HBx microenvironment. Incidentally, the inhibition of PLK1 had no influence on USP37 protein levels in the HBx over-expressing cells unlike that of CDH1 which was rescued in the presence of PLK1 inhibitor (**Figure S3F in file S1**). Thus, USP37 remains protected from SCF-β-TrCP-mediated ubiquitination despite PLK1 activity. The dichotomy in the action of two E3 ligases targeting USP37 further inspired us to investigate the possibility of direct or indirect interaction between HBx and USP37 which could reveal a bigger role of HBx in protection of USP37.

### HBx interacts with USP37 and translocates it to cytoplasm

**Figure 4:**
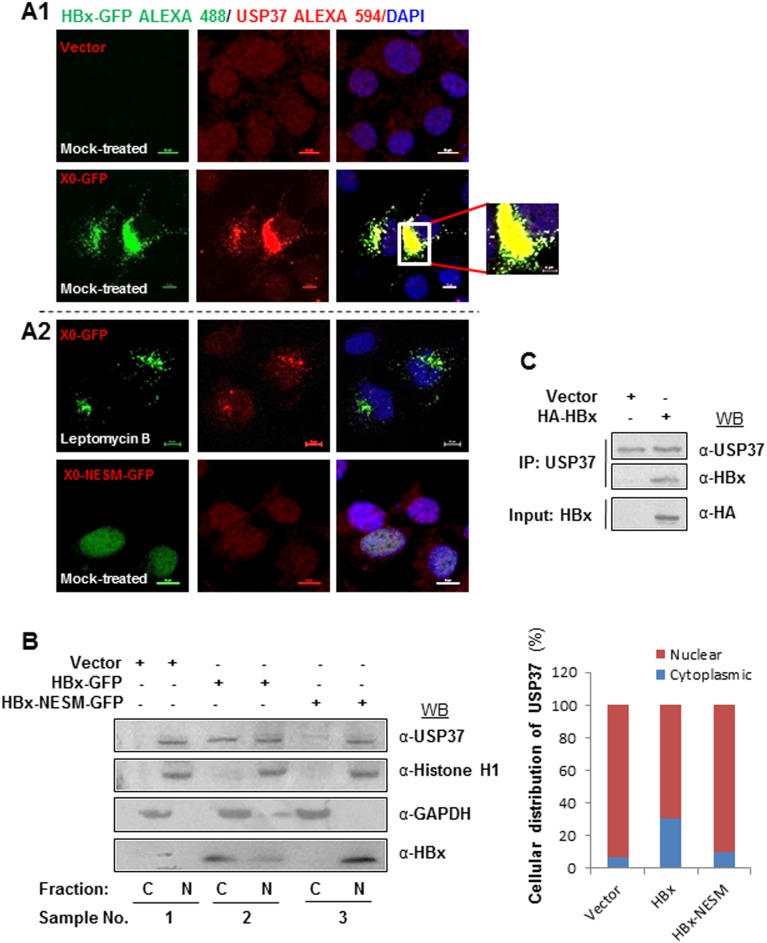
HBx interacts with USP37 and promotes USP37 translocation from Nucleus to cytoplasm. Representative confocal image of mock-treated Huh7 cells transiently expressing Vector, X0-GFP or XO-NESM-GFP constructs and cells expressing X0-GFP construct treated with Leptomycin B (100 nM, 4 h), fixed and stained with anti-HBx and anti-USP37 primary antibody and corresponding fluorescent-labelled secondary antibody. Nuclei were counterstained with DAPI. Images were captured at 60x magnification in Nikon A1R confocal microscope. Scale bar represents 10 µm unless mentioned otherwise. (B) Cytoplasmic-nuclear fractionation was performed with cells transfected with vector, HBx-GFP or HBx-NESM-GFP constructs as per the protocol mentioned earlier. The Nuclear (N) and the cytoplasmic (C) fractions of cells were western blotted with USP37, HBx, Histone H1 and GAPDH antibodies. (C) USP37 was immunoprecipitated from cell lysate of Huh7 cells transfected with Vector or HA-HBx. Immuno-complexes were separated on SDS-PAGE gel and were immunoblotted using USP37 and HBx anitibody. Input was probed with HA antibody.

USP37 has been reported previously to majorly localize in the nucleoplasm [32]. However, we observed that under HBx environment, USP37 translocated in the peri-nuclear-cytoplasmic region and co-localized with HBx (Pearson’s co-efficient of correlation = 0.994292; Mander’s co-efficient of correlation = 0.658119; n = 25). In contrast, USP37 was primarily nuclear in the control cells (**Figure 4A** **1**). These findings were further substantiated by nuclear and cytoplasmic fractionation of cells transfected with HBx and its hairpin construct (**Figure S4A in file S1**). Interestingly, the cytoplasmic compartmentalization of USP37 could be reversed in the presence of HBx sh-RNA (X-E). It has been reported earlier nuclear export signal of HBx is responsible for its nuclear export in a cellular milieu [21]. Moreover, mutation of its two Leucine residues to Alanine (L98A, L100A) in ‘NES element’ renders it nucleus bound [21]. The nuclear export of HBx was also sensitive to Leptomycin B treatment (CRM1/XPO dependent nuclear transport inhibitor) [21]. Intriguingly, USP37 migration to cytoplasm in the presence of HBx, was also found to be sensitive to Leptomycin B treatment. This observation prompted us to investigate if HBx acted as chaperone for USP37. As expected, over-expression of the nuclear export signal mutant of HBx (HBx-NESM-GFP) abolished the nuclear export of USP37 (**Figure 4A** **2**). Consistent with this, our subcellular fractionation studies revealed that while USP37 was present both in the nucleus as well as in cytoplasm in the presence of HBx, it was majorly nuclear in the HBx-NESM transfected cells (**Figure 4B**). Since, HBx and USP37 co-localized in cells, we next examined the possibility of a physical interaction between HBx and USP37. Our co-immunoprecipitation studies revealed that the purified recombinant HBx-MBP fusion protein interacted with endogenous USP37 present in HEK293T cell lysates but not with MBP (**Figure S4B in file S1**). The HBx-USP37 interaction was further confirmed by co-immunoprecipitation of HBx by Flag-tagged USP37 from cells co-transfected with USP37 and HBx (**Figure S4C in file S1**). In addition, HBx was found to interact with endogenously expressed USP37 in cells overexpressing HBx (**Figure 4C**).

### Nuclear export of USP37 rescues it from ubiquitination and proteasomal degradation

**Figure 5:**
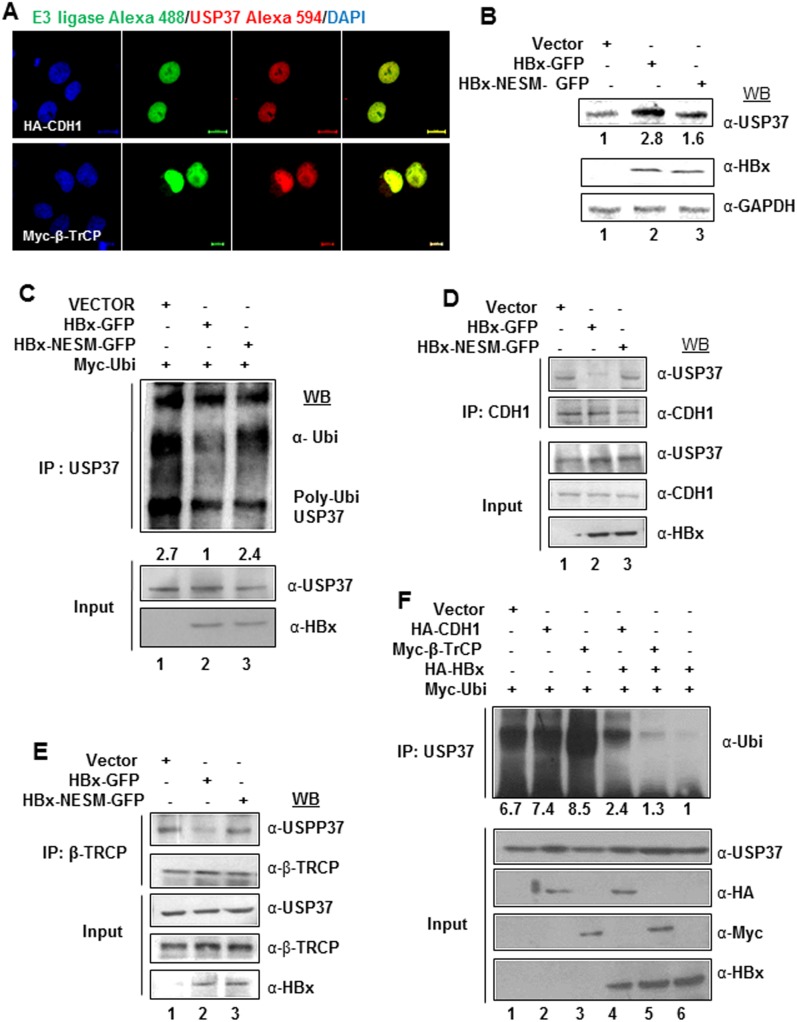
HBx spatially isolate USP37 from its E3 ligases to inhibit USP37 ubiquitination. (A) Representative confocal image of Huh7 cells transiently expressing HA-CDH1 or Myc-β-TrCP constructs, fixed and stained with α-CDH1 or α-β-TrCP and α-USP37 primary antibody and corresponding fluorescent-labelled secondary antibody. Nuclei were counterstained with DAPI. Images were captured at 60x magnification in Nikon A1R confocal microscope. Scale bar represents 10 µm unless mentioned otherwise. (B) Cell extracts from cells transfected with Vector, HBx-GFP and HBx-NESM-GFP as indicated, were separated on SDS-PAGE gel and western blotted with antibody against USP37. GAPDH was used as loading control. (C) Ubiquitination assay was performed by immunoprecipitating cell lysates from Huh7 cells ectopically expressing Vector and Myc-Ubi, HBx-GFP and Myc-Ubi or HBx-NESM-GFP and Myc-Ubi (2.5 ug DNA of each recombinant was to make a total of 5 ug DNA was transfected in 100 mm Dish); and treated with 20 µM MG132 for 6 h, with USP37 antibody. Immuno-complexes were eluted and western blotted with α-Ubiquitin antibody. (D) Cell extracts from Huh7 cells transiently transfected with Vector, HBx-GFP or HBx-NESM-GFP were immunoprecipitated with α-CDH1 antibody. Immuno-complexes were western blotted with α-USP37 and α-CDH1 antibodies. (E) Cell extracts from Huh7 cells transiently transfected with Vector, HBx-GFP or HBx-NESM-GFP constructs were immunoprecipitated with α-β-TrCP antibody. Immuno-complexes were western blotted with α-USP37 and α-β-TrCP antibodies. (F) Ubiquitination assay was performed by immunoprecipitating cell lysates from Huh7 cells transiently expressing Vector and Myc-Ubi; HA-CDH1 and Myc-Ubi; Myc-β-TrCP and Myc-Ubi; HA-X0, HA-CDH1 and Myc-Ubi; HA-X0, Myc-β-TrCP and Myc-Ubi or HA-X0 and Myc-Ubi (Each 100 mm dish was transfected with 2 ug DNA of indicated plasmids to make a total of 6 ug DNA transfected per dish. Where co-expression of two plasmids (total DNA-4 ug) is indicated the transfection was normalized with 2 ug of Vector construct to ensure equal transfection of DNA); and treated with 20 µM MG132 for 6 h, with USP37 antibody. Immuno-complexes were eluted and western blotted with α-Ubiquitin antibody.

Earlier studies have identified nucleus as the site for the E3 ubiquitin ligase activity of CDH1 and β-TrCP [33]–[38]. Since, USP37 is targeted by both these ubiquitin ligases and HBx chaperoned USP37 out of the nucleus, we wondered if it was safeguard mechanism to prevent the ubiquitination and degradation of USP37. Our, Confocal experiments confirmed that bulk of USP37 interacted with its E3 ligases- CDH1 (Pearson’s co-efficient of correlation = 0.984381; Mander’s co-efficient of correlation = 0.581855; n = 25) and β-TrCP (Pearson’s co-efficient of Correlation = 0.979543; Mander’s co-efficient of correlation = 0.557357; n = 25) inside the nucleus (**Figure 5A**). Not surprisingly, over-expression of HBx-NESM mutant did not confer stability to USP37 protein (**Figure 5B**) owing to its inability to interfere with its ubiquitination (**Figure 5C**). As expected, overexpression of HBx unlike NESM mutant abrogated the interaction of USP37 with its cognate E3 ligases- CDH1 and β-TrCP (**Figure 5D and 5E**). Moreover, our ubiquitination assay confirmed that HBx did not interfere with the ubiquitination of USP37 by CDH1 and β-TrCP (**Figure 5F**). These observations suggested that HBx could stabilize USP37 by protecting it from ubiquitination and subsequent proteasomal degradation.

## Discussion

HBx is a bonafide oncoprotein of HBV that extends its influence over a range of host cell functions like cell cycle progression, signaling pathways, DNA damage response, gene expression and regulation of ubiquitin-proteasomal system to facilitate virus-mediated carcinogenesis [11]. Not surprisingly, Ubiquitin ligases are increasingly being recognized as being instrumental in oncogenesis. HBx is now also known to manipulate few E3 ligases and their adaptors like SCF/S-phase kinase associated protein 2 (SCF/Skp2), SCF/F-box/WD repeat-containing protein 7 α (SCF/Fbw7α), SCF/Suppressor of cytokine signaling 3 (SCF/SOCS3), Adenomatosis Polyposis Coli, APC/CDC20 or APC/CDH1 [39]. Ironically, the interaction of HBx with cellular deubiquitinases has not studied. Recently, deubiquitinase USP37 has been recognized as a cell cycle regulator which reverses APC/CDH1 mediated ubiquitination of cyclin A to promote S phase entry of cells [6]. Besides, its normal functions, USP37 is gaining relevance in context of cancer development. For instance, increased USP37 expression is correlated with poor prognosis in non-small cell lung cancer [8]. USP37 also plays a role in stabilizing tumor suppressor p27 in medulloblastoma cells and promyelocytic leukemia zinc finger and retinoic acid receptor alpha (PLZF-RARA) oncogenic fusion protein in Acute promyelocytic leukemia [9], [10]. As HBx has been shown to stabilze cyclin A in order to potentiate CDK2 activity [13], we wondered if HBx engaged USP37 in conferring intracellular stability to cyclin A.

The present study showed that HBx involved USP37 in the stabilization of Cyclin A and the effect could be reversed in the presence of USP37-DD. Furthermore, FACS analysis revealed that USP37 played a crucial role in HBx-mediated deregulation of cell cycle by accelerating the S phase entry and the effect could be reversed in the presence of USP37-DD mutant. Earlier reports have suggested that stable expression of Cyclin A inside the cells can cause cell cycle arrest or apoptosis due to checkpoint activation [40]. In contrast, our Brd-U and MTT assays revealed that cells co-transfected with HBx and USP37 showed improved cellular viability and active DNA replication. As HBx has been documented to overcome cell cycle checkpoints in order to support unfettered cell cycle progression [31], [41], the higher viability of cells co-transfected with HBx and USP37 could be attributed to HBx-mediated checkpoint inactivation.

HBx was also found to stimulate the gene transcription and protein expression of USP37. Since, three broad mechanisms, viz., - transcription, translation and post-translational modifications regulate the intracellular levels of protein, we wondered if the transcriptional activation USP37 gene could influence USP37 protein levels in the HBx transfected cells. In agreement with earlier reports, the elevated transactivation of USP37 gene relates to the heightened activity of E2F1 transcription factor under the HBx microenvironment [6], [27]. Not surprisingly, transactivation mutant of E2F1, E2F1 1-374 (ΔC) is unable to stimulate the expression of USP37 gene. Ironically, the co-expression of neither E2F1 nor E2F1 1-374 (ΔC) along with HBx did not affect the basal expression of USP37 protein suggesting the involvement of other regulatory mechanisms in this process. However, we do not exclude the possibility of enhanced USP37 expression to be a consequence of increased E2F1 activity in cells rather than by HBx alone (Fig. 2C and Fig. S2F in File S1).

Not surprisingly, we observed a marked increase in the stability and intracellular accumulation of USP37 protein under the HBx microenvironment. We found that the elevated stability of USP37 was an outcome of its escape from proteasomal degradation. HBx directly attenuated the ubiquitination and subsequent proteasomal degradation of USP37 which could be reversed by RNA interference against HBx. As HBx could effectively prevent the ubiquitination of USP37, we assessed the regulation of its E3 ubiquitin ligases- CDH1 and β-TrCP in the presence of HBx. It has been shown recently that the stabilization of replication licensing factor CDC6 by HBx is a cumulative effect of down-regulation of its E3 ubiquitin ligase- CDH1 and increase in its post-translational modification (phosphorylation) by CDK2, leading to subdued ubiquitination by CDH1 [27]. Quite similarly, the down-regulation of CDH1 in the presence of HBx conferred stability to USP37 just as CDC6 and emerged as a strategy to stabilize USP37. A recent study implicates the phosphorylation of Ser-628 of USP37 to protect it from CDH1 mediated ubiquitination [6]. Further, inhibition of CDK2 activity in HBx expressing cells, resulted in a decrease of not only phosphorylated USP37 but also total USP37 protein levels. Thus, enhanced Cyclin A/CDK2 activity under HBx microenvironment [12] ensured the protection of USP37 from CDH1-catalysed ubiquitination. As Emi1, a pseudosubstrate of APC/CDH1 complex that competitively prevents the degradation of another substrate Skp2 of CDH1 [29] and is found to be upregulated in HCC [42], we also investigated the possibility of recruitment of Emi1 by HBx in stabilizing USP37. Ironically, Emi1 overexpression did not lead to USP37 accumulation thereby mitigating the possible role of Emi1 in HBx-mediated USP37 stabilization.

Having established two core mechanisms, i.e., down-regulation of CDH1 levels and post-translational modification of USP37 by Cyclin A/CDK2 complex in stabilizing USP37 from CDH1 mediated ubiquitination we shifted our focus to the regulation of second E3 ubiquitin ligase of USP37 - β-TrCP, by HBx. Paradoxically, despite the conspicuous up-regulation of β-TrCP (both mRNA and protein levels) in the presence HBx, USP37 remained resilient to the onslaught of SCF/β-TrCP complex similar to β-catenin which is rescued from SCF/β-TrCP complex by inhibition of Glycogen Synthase Kinase 3β (GSK3β) activity by HBx [43]. Further, despite HBx-stimulated upsurge in PLK1 activity [31], which facilitates the recognition of USP37 by β-TrCP, USP37 remained stable under the HBx microenvironment. Nevertheless, the Plk1 mediated phosphorylation and down-regulation of the levels of CDH1 protein reinforced our observations on the protective effect of HBx on USP37.

Venturing further we explored the possibility of a physical interaction between HBx and USP37. We identified USP37 as a novel interactor of HBx. Interestingly, in contrast to the earlier reports on nuclear distribution of USP37 [32], we found that USP37 co-localized with HBx in the cytoplasm through a chaperoning mechanism. Many recent studies have highlighted the significant role of E3 ubiquitin ligases, deubiquitinases or substrate translocation between cell compartments, leading to substrate stability motivated us to explore the effect of HBx-driven exodus of USP37 from the nucleus vis-à-vis its intracellular stability. The logic was based on some interesting observations, such as, β-TrCP committed to degrade the GSK3β phosphorylated DNA methyl transferase 1 (DNMT1) is prevented by tobacco-specific carcinogen NNK-induced β-TrCP translocation to the cytoplasm with the help of heterogeneous nuclear ribonucleoprotein U (hnRNP-U) [38]. Yet another report suggests that in chondrocytes, β-catenin is rescued from its cytoplasmic degradation by SCF/β-TrCP upon its SMAD3-SMAD4 mediated translocation to the nucleus triggered by TGF-β [44]. A recent study where another DUB USP7, originally a nuclear protein present inside the PML (promyelocytic leukemia) bodies is tethered to the cytoplasm by Infected Cell protein 0 (ICP0) oncoprotein of Herpes Simplex Virus where it deubiquitinates and stabilizes TNF receptor associated Factor 6 (TRAF6) and Iκκ-γ [45], also beautifully illustrate this paradigm. Since, CDH1 and β-TrCP are reported to ubiquitinate plethora of substrates inside the nucleus [33]–[38] and in the present study were found to co-localize with USP37 inside the nucleus, the HBx-mediated compartment shuffling of USP37 appeared to be a novel mechanism to ensure USP37 indemnity. Further, ubiquitination, sub-cellular fractionation and immunoprecipitation assays using wild type and NES mutant of HBx, established that HBx-mediated nuclear export of USP37 indeed prevents its ubiquitination by spatially segregating USP37 from its E3 ligases- CDH1 and β-TrCP. Thus, the present study shows that HBx has a profound influence over the expression and intracellular distribution of USP37 which may be a part of the elaborate mechanism involved in cell cycle deregulation and cellular transformation. Whether or not the HBx-USP37 axis operates *in vivo* cannot be said with certainty and thus, would require further investigation. This study has relied on the cell culture-based system where HBx was co-expressed along with USP37-DD or USP37 either in immortalized human hepatocytes or in hepatoma Huh7 cells to elucidate the oncogenic cooperation between HBx and USP37. Therefore, it will be desirable to substantiate these findings in primary hepatocytes, USP37 null cells as well as in experimental animal models of viral HBx. Nevertheless, it appears that HBx could employ cellular USP37 as a novel strategy to deregulate cell cycle and induce cell transformation.

## Supporting Information

File S1 **Supporting files.** This file contains Table S1, Figure S1, Figure S2, Figure S3, and Figure S4. **Figure S1**, Status of cell cycle regulators and cell viability under different experimental conditions. **Figure S2**, Validation of HBx expression and stimulation of USP37 by HBx. **Figure S3**, Regulation of USP37 under HBx microenvironment. **Figure S4**, Intracellular distribution of USP37 and its interaction with HBx. **Table S1**, tabulates primer names and sequences used for RT PCR.
(PDF)
