# Expression of Concern: The HBx Oncoprotein of Hepatitis B Virus Deregulates the Cell Cycle by Promoting the Intracellular Accumulation and Re-Compartmentalization of the Cellular Deubiquitinase USP37

**DOI:** 10.1371/journal.pone.0293102

**Published:** 2023-10-12

**Authors:** The PLOS ONE Editors

Following the publication of this article [1], concerns were raised regarding Fig 2.

Specifically,

- In Fig 2D, lanes 2 and 3 of the α-USP37 panel appear similar to lanes 2 and 3 of the α-GAPDH panel flipped horizontally and rotated.
- In Fig 2E, there appear to be discontinuities in the background between the lanes in the IP: USP37 panel.

In reviewing this matter, PLOS noted that the primary data were not provided with the published article [1], contrary to the Data Availability statement. The corresponding author stated that the data underlying the results in this article are no longer available, and so the article [1] does not comply with the PLOS Data Availability policy.

In relation to Fig 2D, the corresponding author acknowledged that an incorrect image may have been used during figure preparation. In the absence of original underlying data, this issue cannot be resolved.

For Fig 2E, the corresponding author stated that discontinuities could have occurred due to the experimental conditions. This issue cannot be fully clarified without the original underlying data.

The *PLOS ONE* Editors issue this Expression of Concern to notify readers of the unresolved image concerns and the unavailability of the primary data.
