# Supplementary material for: The HBx Oncoprotein of Hepatitis B Virus Deregulates the Cell Cycle by Promoting the Intracellular Accumulation and Re-Compartmentalization of the Cellular Deubiquitinase USP37
Source: PLoS One. 2014 Oct 27;9(10):e111256. doi: 10.1371/journal.pone.0111256 (PMC4210131; doi:10.1371/journal.pone.0111256)
Supplement: File S1 — Supporting files. This file contains Table S1, Figure S1, Figure S2, Figure S3, and Figure S4. Figure S1, Status of cell cycle regulators and cell viability under different experimental conditions. Figure S2, Validation of HBx expression and stimulation of USP37 by HBx. Figure S3, Regulation of USP37 under HBx microenvironment. Figure S4, Intracellular distribution of USP37 and its interaction with HBx. Table S1, tabulates primer names and sequences used for RT PCR. (PDF) [file pone.0111256.s001.pdf]

# Supplementary Information

## Table S1

| Primers for RT-PCR | Sequence 5'-->3' |
|---|---|
| **Cdh1 Fwd primer** | AGATCTCCAAGATCCCCTTCA |
| **Cdh1 Rev primer** | CCTCCAACATGGACAGCTTCT |
| **USP37 Fwd primer** | CCAGTGGAGCGAAACAAAGC |
| **USP37 Rev primer** | CCTCTGCATCCTTACTTGGTACT |
| **B-trcp Fwd primer** | ATGCAAGCGAATTCTCACAGG |
| **B-trcp Rev primer** | GGAACGATCTTTGGAGCAGGT |

Fwd, forward; Rev, reverse

# Figure S1

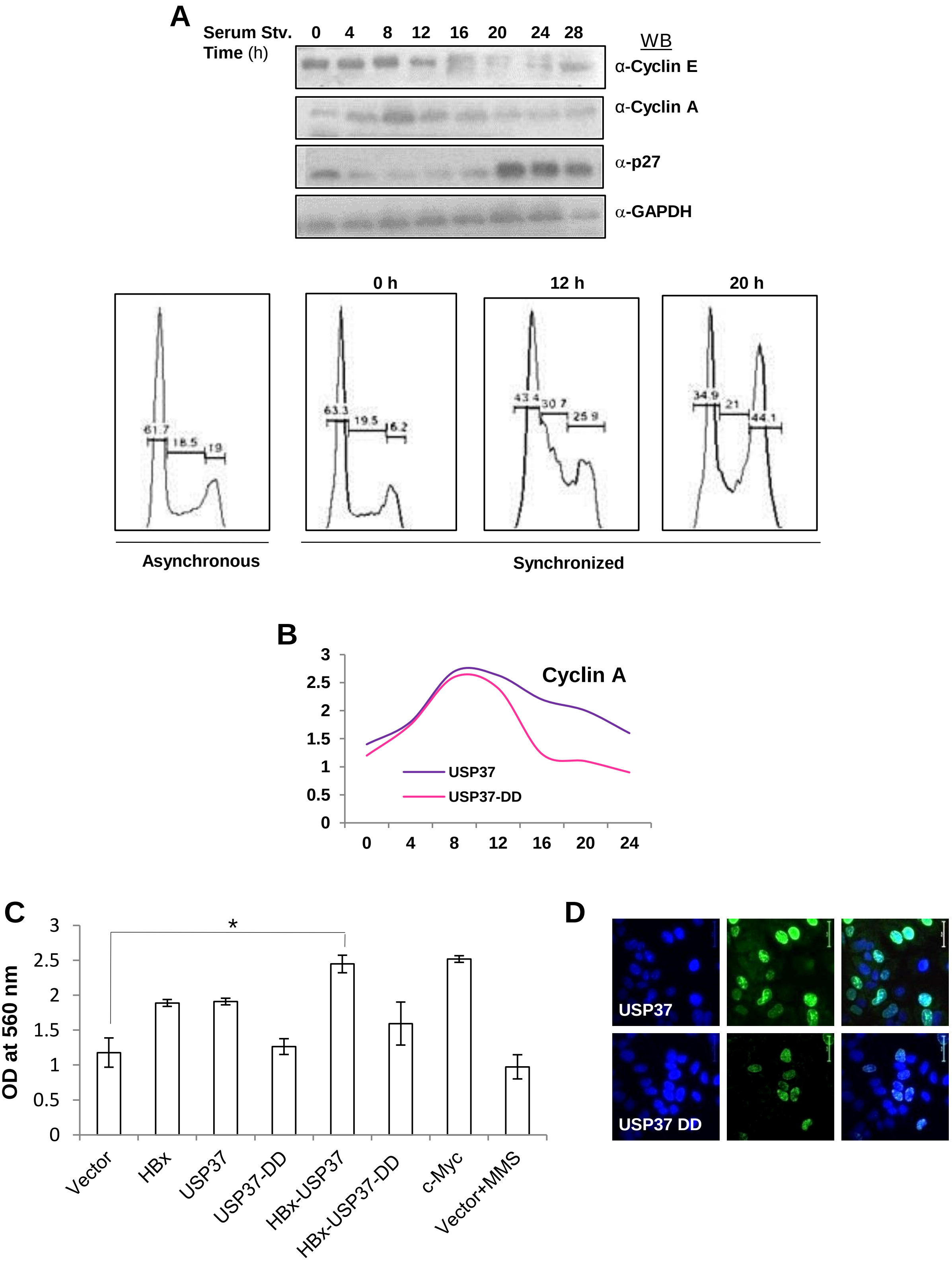
A
Serum Stv. Time (h)
0 4 8 12 16 20 24 28
WB
α-Cyclin E
α-Cyclin A
α-p27
α-GAPDH
0 h
12 h
20 h
61.7
18.5
19
63.3
19.5
6.2
43.4
30.7
25.9
34.9
21
44.1
Asynchronous
Synchronized
B
Cyclin A
USP37
USP37-DD
0 4 8 12 16 20 24
0 0.5 1 1.5 2 2.5 3
C
OD at 560 nm
*
Vector
HBx
USP37
USP37-DD
HBx-USP37
HBx-USP37-DD
c-Myc
Vector+MMS
D
USP37
USP37 DD

**Figure S1. Status of cell cycle regulators and cell viability under different experimental conditions.** (A) IHH cells were serum starved for 72 h followed by serum stimulation and harvesting at indicated time points. Cell cycle analysis was performed by flow cytometry after staining with propidium iodide . The expression of Cyclin E, Cyclin A, p27 and GAPDH was analyzed by western blotting. (B) IHH cells were transfected with Flag-USP37 and Flag-USP37-DD and the levels of Cyclin A were measured at indicated time periods. (C) Huh7 cells were transfected with control vector; HBx; Flag-USP37; Flag-USP37-DD; HBx and Flag-USP37 and HBx and Flag-USP37-DD and cell viability was measured by MTT assay. Cells transfected with HA-Myc and vector and treated with Methyl Methane Sulphonate (97% w/v) at 0.1% v/v for 30 min were used as positive and negative control, respectively. Data (bar diagrams) are shown as mean ± SD of three independent observations . * represents statistically significant difference of $p<0.005$. (D) Huh7 cells transfected with Flag-USP37 and Flag-USP37-DD were analyzed for Brd-U incorporation and confocal imaging. Scale bar represents 50μm.

# Figure S2

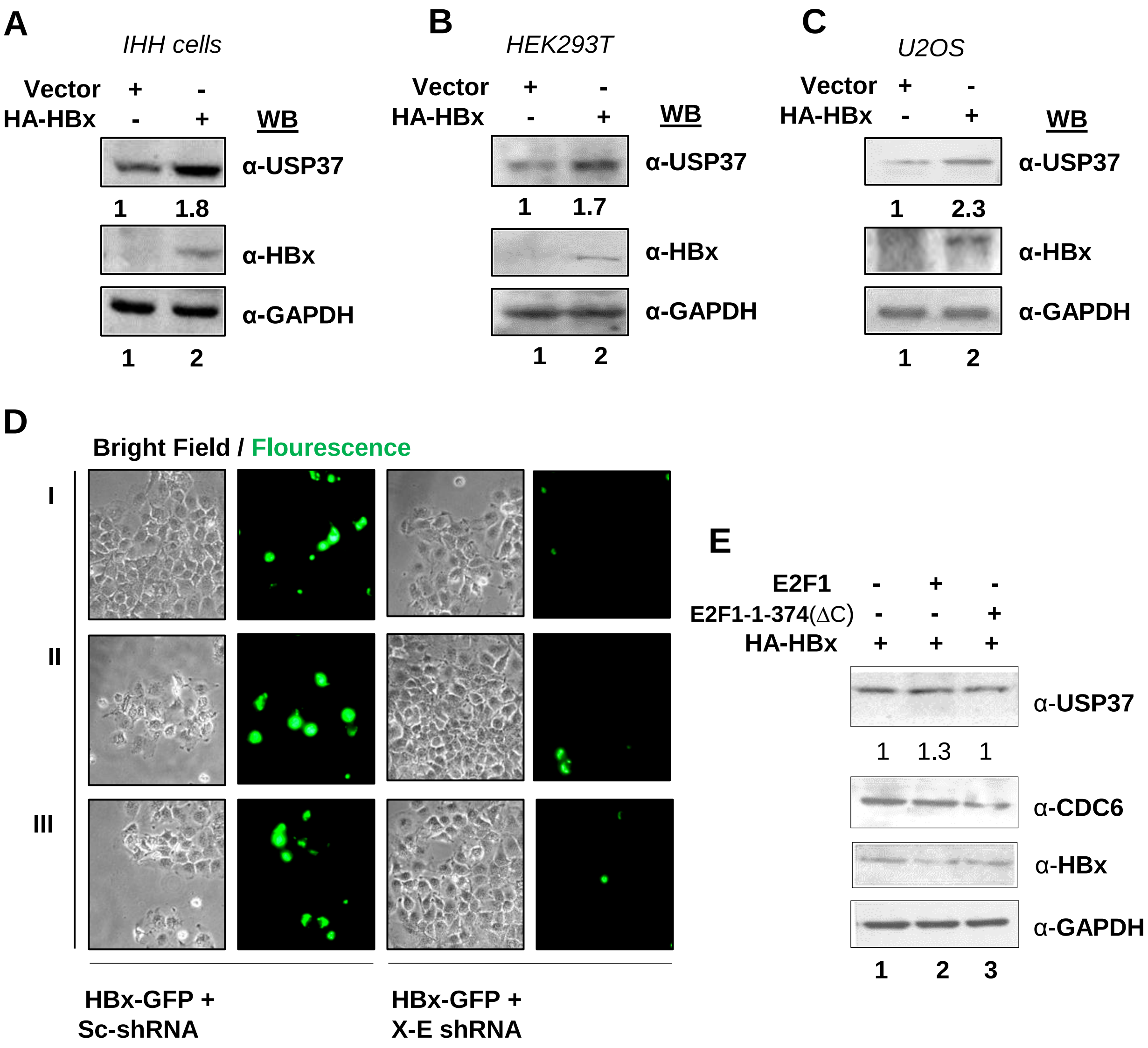
A
IHH cells
Vector + -
HA-HBx - +
WB
α-USP37
1 1.8
α-HBx
α-GAPDH
1 2
B
HEK293T
Vector + -
HA-HBx - +
WB
α-USP37
1 1.7
α-HBx
α-GAPDH
1 2
C
U2OS
Vector + -
HA-HBx - +
WB
α-USP37
1 2.3
α-HBx
α-GAPDH
1 2
D
Bright Field / Flourescence
I
II
III
HBx-GFP +
Sc-shRNA
HBx-GFP +
X-E shRNA
E
E2F1 - + -
E2F1-1-374(ΔC) - - +
HA-HBx + + +
α-USP37
1 1.3 1
α-CDC6
α-HBx
α-GAPDH
1 2 3

**Figure S2. Validation of HBx expression and stimulation of USP37 by HBx.** IHH (A), HEK293 (B) and U2OS cells (C) were transfected with vector control or HA-HBx expression vector and the levels of USP37 and HBx were measured by western blotting. GAPDH was used for normalization of protein levels. (D) Huh7 cells were transfected with HBx-GFP along with Sc-shRNA or X-E shRNA. Bright field and fluorescence images of cell s (panels I-III) showing the expression HBx-GFP fusion protein. (E) Huh7 cells were transfected with HA-HBx (along with E2F1 or E2F1-Δ1-374 . And the cell lysates were western blotted with α-USP37 and α-CDC6 antibodies. GAPDH was used as a loading control.

Figure S3

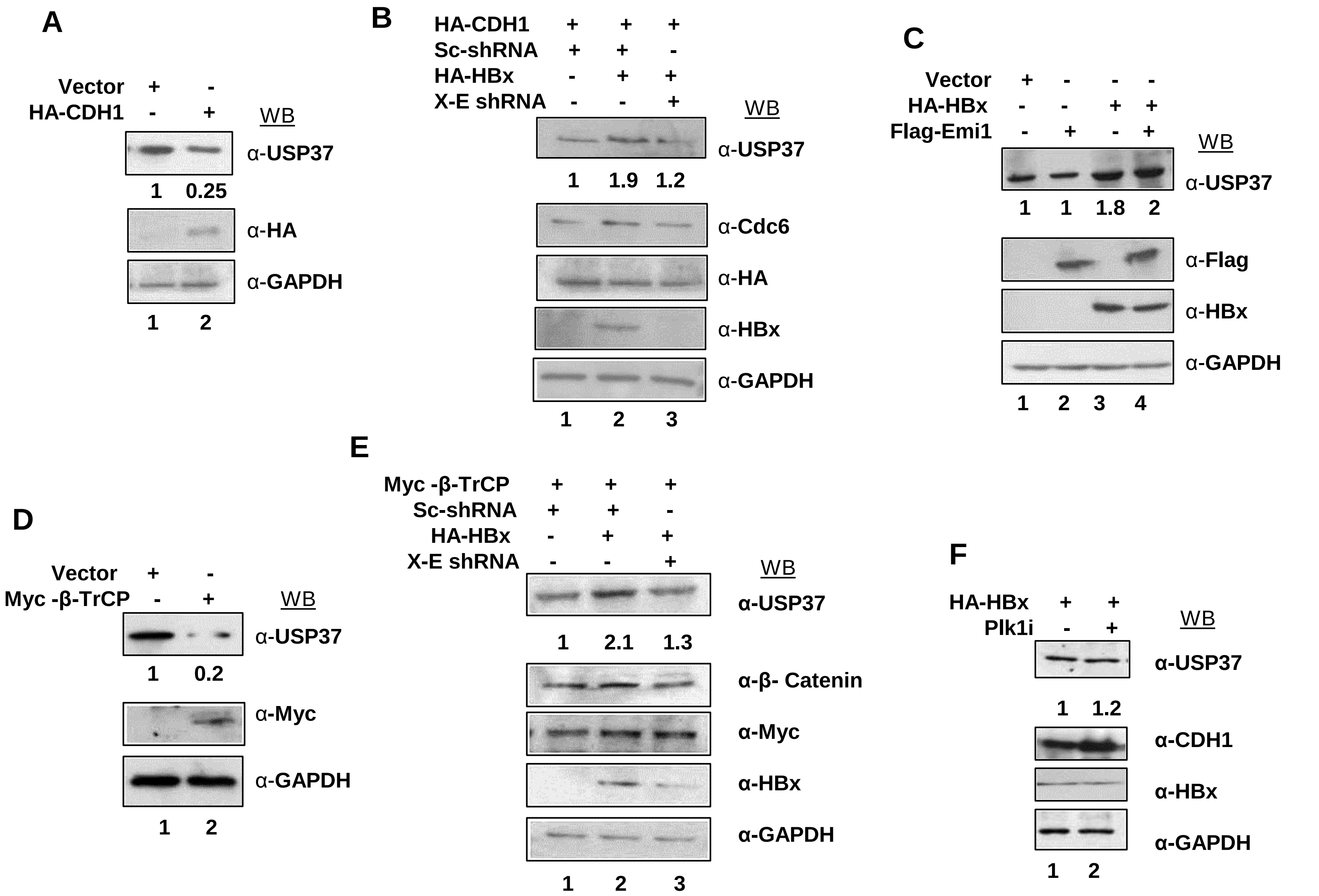
A
Vector + -
HA-CDH1 - +
WB
α-USP37
1 0.25
α-HA
α-GAPDH
1 2
B
HA-CDH1 + + +
Sc-shRNA + + -
HA-HBx - + +
X-E shRNA - - +
WB
α-USP37
1 1.9 1.2
α-Cdc6
α-HA
α-HBx
α-GAPDH
1 2 3
C
Vector + - - -
HA-HBx - - + +
Flag-Emi1 - + - +
WB
α-USP37
1 1 1.8 2
α-Flag
α-HBx
α-GAPDH
1 2 3 4
D
Vector + -
Myc -β-TrCP - +
WB
α-USP37
1 0.2
α-Myc
α-GAPDH
1 2
E
Myc -β-TrCP + + +
Sc-shRNA + + -
HA-HBx - + +
X-E shRNA - - +
WB
α-USP37
1 2.1 1.3
α-β- Catenin
α-Myc
α-HBx
α-GAPDH
1 2 3
F
HA-HBx + +
Plk1i - +
WB
α-USP37
1 1.2
α-CDH1
α-HBx
α-GAPDH
1 2

**Figure S3. Regulation of USP37 under HBx microenvironment.** Huh7 cells were transfected with indicated recombinants and western blotted for the expression of specific antigens: (A) transfection with vector or HA-CDH1 constructs and immunoblotting for USP37; (B) transfection with HA-CDH1, HA-HBx, Sc-shRNA and/or Sc-shRNA and probing with α-USP37, α-Cdc6 , α-HBx and α-HA antibodies; (C) transfection with vector, Flag-Emi1 and HA-HBx and western blotting with α-USP37, α-Flag and α-HBx antibodies; (D) transfection with vector and Myc-β-TrcP constructs and western blotting for USP37; (E) transfection with combinations of Myc-β-TrcP, HA-HBx, Sc-shRNA and X-E-shRNA as indicated and western blotted with α-USP37 and α-β-catenin antibodies; (F) transfection with HBx, treated with PLK1 inhibitor SBE13 hydrochloride (100µM) for 8h, and western blotted for USP37 and CDH1. GAPDH levels were used for normalization in the above panels.

Figure S4

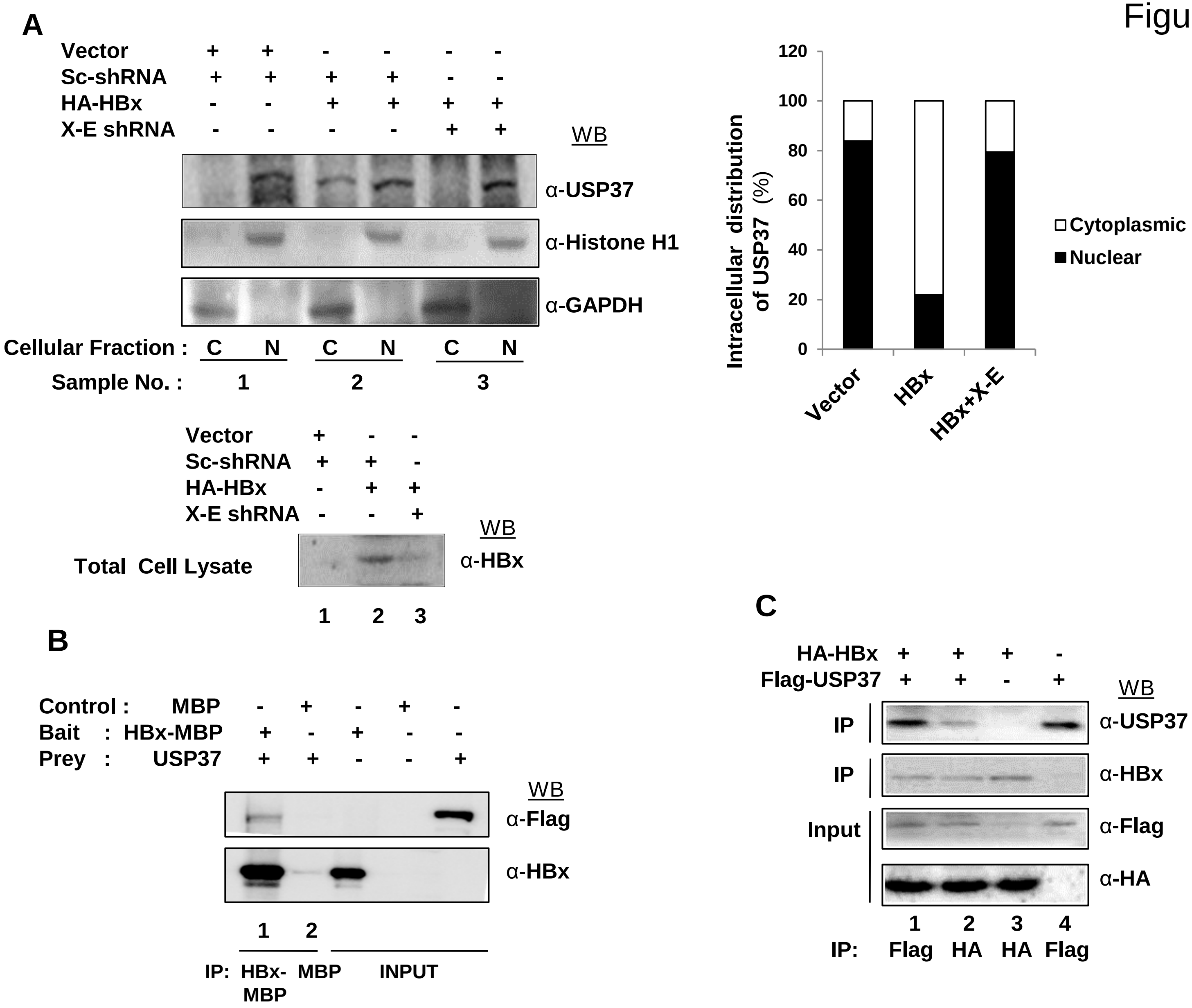

A
Vector + + - - - -
Sc-shRNA + + + + - -
HA-HBx - - + + + +
X-E shRNA - - - - + +
WB
α-USP37
α-Histone H1
α-GAPDH
Cellular Fraction : C N C N C N
Sample No. : 1 2 3
Intracellular distribution of USP37 (%)
120
100
80
60
40
20
0
Vector
HBx
HBx+X-E
Cytoplasmic
Nuclear
Vector + - -
Sc-shRNA + + -
HA-HBx - + +
X-E shRNA - - +
WB
Total Cell Lysate
α-HBx
1 2 3
B
Control : MBP - + - + -
Bait : HBx-MBP + - + - -
Prey : USP37 + + - - +
WB
α-Flag
α-HBx
1 2
IP: HBx-MBP MBP INPUT
C
HA-HBx + + + -
Flag-USP37 + + - +
WB
IP
α-USP37
IP
α-HBx
Input
α-Flag
α-HA
1 2 3 4
IP: Flag HA HA Flag

**Figure S4. Intracellular distribution of USP37 and its interaction with HBx.** (A) HEK293T cells were transfected with vector, HA-HBx, Scrambled (Sc)-shRNA and X-E shRNA constructs, and the nuclear (N) and cytoplasmic (C) fractions of cells were western blotted with USP37, Histone H1, HBx and GAPDH antibodies. (B) Amylose beads were bound with recombinant MBP (Control) or HBx-MBP fusion proteins (Prey) and incubated with cell lysates from HEK293T cells transfected with vector or Flag-USP37 (Bait). Eluted immuno-complexes were western blotted with α-Flag and α-HBx antibodies. (C) Cell lysates of Huh7 cells overexpressing HA-HBx and Flag-USP37 were immunoprecipitated using α-Flag and α-HA antibodies as indicated. Eluted immuno-complexes were western blotted with α-USP37, α-HBx, α-HA and α-Flag antibodies.
